# Spondin-1 Inhibits Odontoblastic Differentiation of Human Dental Pulp Stem Cells

**DOI:** 10.3390/biom16060769

**Published:** 2026-05-23

**Authors:** Bara Mardini, Hideki Sugii, Koudai Tashita, Mhd Safwan Albougha, Serina Soeno, Ryosuke Tachibana, Ömer Tarık Özdemir, Kanon Nasu, Sayuri Hamano, Hidefumi Maeda

**Affiliations:** 1 Department of Endodontology and Operative Dentistry, Faculty of Dental Science, Kyushu University, Fukuoka 812-8582, Japan; bara.mardini@dent.kyushu-u.ac.jp (B.M.);; 2 Department of Endodontology, Kyushu University Hospital, 3-1-1 Maidashi, Higashi-ku, Fukuoka 812-8582, Japan; 3 Department of Pedodontics, Faculty of Dentistry, Istanbul University, Istanbul 34810, Turkey

**Keywords:** SPON1, F-spondin, human dental pulp stem cells (HDPSCs), odontoblastic differentiation, reparative dentin formation, mineralization, Wnt/β-catenin signaling

## Abstract

Reparative dentin formation is a defensive response that restores a mineralized barrier to protect the dental pulp following various stimuli, such as bacterial invasion, tooth preparation, or restorative materials. However, reparative dentin is limited, and to avoid pathological calcification or pulp canal obliteration, mineral deposition must be restricted to the injured area and temporally restrained once the barrier is reestablished. This suggests the existence of negative regulators that can halt odontoblastic differentiation; however, such inhibitory regulators remain incompletely defined. Spondin-1 (SPON1) is an extracellular matrix protein known to regulate bone homeostasis and act as a negative regulator of bone mass; however, the effects of SPON1 on odontoblastic differentiation of dental pulp stem cells (DPSCs) remain unclear. This study aimed to analyze the effects of SPON1 on odontoblastic differentiation of human DPSCs (HDPSCs). SPON1 was expressed in the odontoblastic layer and dental pulp tissue, and its expression was significantly decreased at the beginning of reparative dentin formation in rats. Treatment with SPON1 inhibited odontoblastic differentiation of HDPSCs by blocking the expression of non-phosphorylated β-catenin, while neutralizing SPON1 significantly enhanced odontoblastic differentiation of HDPSCs. These findings suggest that SPON1 functions as a negative regulator of odontoblastic differentiation during reparative dentin formation.

## 1. Introduction

Dentin is a complex mineralized tissue that is formed by highly specialized cells called odontoblasts, which secrete an extracellular matrix (ECM) comprising collagen fibers and non-collagenous proteins that serve as a scaffold for subsequent mineralization [1]. Dentin provides the primary structural support to the tooth and plays a critical role in maintaining pulp vitality. The loss of dentin, most commonly as a result of dental caries or traumatic injury, may lead to endodontic treatment. However, endodontic treatment is associated with a significantly increased risk of tooth extraction because of vertical root fractures caused by thinning of the tooth root structure [2]. To achieve long-term tooth preservation, it is therefore important to minimize the need for endodontic treatment by protecting the dentin.

Dentin formation during the lifetime of a vital tooth is described as primary, secondary, or tertiary dentin. Primary dentin is formed prior to tooth eruption until the tooth becomes functional, while secondary dentin starts to be deposited when contacts between antagonistic cusps are established, and continues throughout life at a much slower rate [1]. In contrast, tertiary dentin is formed locally in response to external stimuli such as carious lesions, abrasion, attrition, cavity preparation, or traumatic injury, and acts as a protective barrier for the underlying pulp tissue. The biological mechanisms underlying tertiary dentinogenesis vary depending on the severity of the stimulus. The secretion of tertiary dentin under mild to moderate stress is referred to as “reactionary dentin”, in which the odontoblast layer is preserved. In more severe conditions, however, odontoblast survival may be compromised, and the healing process is more complex in such cases, entailing “reparative dentin formation” produced by newly formed odontoblast-like cells differentiated specifically from dental pulp stem cells (DPSCs) [3,4].

Notably, reparative dentin is limited, and mineral deposition must be restricted to the injured area and temporally restrained once the barrier is reestablished to avoid pathological calcification or pulp canal obliteration [5,6]. This suggests that, in addition to pro-differentiation signals, the presence of negative regulatory mechanisms is required to fine-tune odontoblastic differentiation and prevent excessive mineralization. Despite their clinical significance, however, the complex signaling molecules involved in regulating reparative dentin formation remain unclear.

ECM proteins play a major role in determining cell behavior and function by regulating cell adhesion, differentiation, migration, and apoptosis [7], and also play a significant role in mineralization [8]. The ECM protein Spondin-1 (SPON1), also known as F-spondin, is a member of the thrombospondin family encoded by a highly conserved gene. SPON1 is a secreted ECM protein derived from the floor plate of vertebrate embryos and promotes the growth of neural axon cells [9,10]. SPON1 is also expressed in non-neuronal tissues, including the ovary [11], lung [12], and the periodontal ligament [13], suggesting a broader biological role beyond neural development.

In musculoskeletal biology, SPON1 has emerged as a critical regulator of bone metabolism and remodeling. For example, exogenous SPON1 inhibited the longitudinal growth of mouse tibiae [14], and *Spon1*-knockout mice showed a significant increase in trabecular bone mass [15]. These reports suggest that SPON1 may be involved in mineralized tissue homeostasis and may function as a negative regulator of tissue mineralization, acting as a “molecular brake.” Phylogenetic studies have revealed that dentin analogs were evolutionarily very similar to bone, and bone and dentin are known to share common molecular pathways [1]. SPON1 may also regulate odontoblastic differentiation during reparative dentin formation. In addition, activation of Wnt/β-catenin signaling has been reported to positively regulate bone formation, whereas deletion or down-regulation of genes encoding Wnt agonists results in osteopenia [16]. Consistently, canonical Wnt/β-catenin signaling has also been implicated in reparative dentin formation [17,18] and tooth development [19]. We hypothesized that SPON1 may play a role in odontoblastic differentiation of DPSCs during reparative dentin formation; however, no studies have reported on this issue. Therefore, this study aimed to investigate the localization of SPON1 in dental pulp tissue and its effects on odontoblastic differentiation of human DPSCs (HDPSCs), including its possible association with Wnt/β-catenin signaling.

## 2. Materials and Methods

### 2.1. Cell Culture

HDPSCs (DP005), established at Gifu University [20,21], were obtained from RIKEN BioResource Research Center (Tsukuba, Japan) and used at passages three to seven. The cells were maintained in α-Minimum Essential Medium (α-MEM; Gibco, Grand Island, NY, USA) supplemented with 10% fetal bovine serum (FBS; Sigma-Aldrich, St. Louis, MO, USA), 50 U/mL penicillin, and 50 µg/mL streptomycin (FUJIFILM Wako Pure Chemical Corporation, Osaka, Japan), and incubated at 37 °C in 5% CO_2_ (α-MEM/10%FBS).

### 2.2. Odontoblastic Differentiation

Previous studies demonstrated that calcium supplementation promoted the osteo/odontoblastic differentiation and mineralization of HDPSCs [22,23]. We accordingly used calcium chloride (CaCl_2_) to induce odontoblastic differentiation in this study. HDPSCs were seeded at a density of 2 × 10^4^ cells/well in 24-well plates and cultured in α-MEM/10%FBS with 2 mM CaCl_2_ (Nacalai Tesque, Kyoto, Japan). Upon reaching confluence, the cells were maintained in the same medium for odontoblastic induction, and the medium was refreshed every 3 days. The cells were cultured under these conditions for different durations, depending on the experimental endpoint.

### 2.3. Quantitative Reverse Transcription-Polymerase Chain Reaction (qRT-PCR)

For gene expression analysis, HDPSCs were harvested on days 3, 7, and 14, and total RNA was isolated using TRIzol reagent (Invitrogen, Carlsbad, CA, USA) according to the manufacturer’s instructions. For siRNA experiments, total RNA was isolated at the endpoints described in the siRNA transfection section. First-strand cDNA was synthesized from 1 µg of total RNA using an ExScript RT Reagent Kit (Takara Bio Inc., Kusatsu, Japan). Real-time qRT-PCR was performed using THUNDERBIRD™ SYBR™ qPCR Mix (QPS-201; TOYOBO Co., Ltd., Osaka, Japan) using a Thermal Cycler Dice Real-Time System (Takara Bio Inc.). Expression levels of the odontoblast-related markers, dentin matrix protein 1 (*DMP1*), dentin sialophosphoprotein (*DSPP*), nestin (*NESTIN*), lymphoid enhancer-binding factor 1 (*LEF1*), osteocalcin (*OCN*), osteopontin (*OPN*), sclerostin (*SOST*), and F-spondin (*SPON1*) were investigated. Primer sequences were designed using human gene reference sequences obtained from the NCBI GenBank database and verified for specificity using BLAST (version +2.17.0). Each target gene was normalized to β-actin as an internal control. Relative expression levels were calculated using the 2^−ΔΔCt^ method. Primer sequences, annealing temperatures, and product sizes are listed in Supplementary Table.

### 2.4. Mineralization Assay

Mineralized nodule formation was evaluated by Alizarin Red S staining of HDPSCs cultured with odontoblastic differentiation medium (DM) for 7 days. The cells were fixed with 10% neutral-buffered formalin (FUJIFILM Wako Pure Chemical Corporation) for 30 min at room temperature, washed thoroughly with distilled water, and stained with 0.3% Alizarin Red S solution (pH 4.2; Sigma-Aldrich, St. Louis, MO, USA) for 1 h at room temperature. Excess dye was then removed by repeated washing with distilled water. The stained cells were air-dried and imaged under a BZ-9000 microscope (Keyence, Osaka, Japan). Alizarin Red S-positive mineralized areas were quantified using BZ-X Analyzer Software (version 1.1.2.4; Keyence).

### 2.5. Treatment with Recombinant SPON1

HDPSCs were seeded at 3 × 10^4^ cells/well in 24-well plates and cultured in either control medium (CM), DM, or DM supplemented with 100 ng/mL recombinant human SPON1 (R&D Systems, Minneapolis, MN, USA) (DM + SPON1). Phosphate-buffered saline (PBS; Nacalai Tesque) was used as the diluent for recombinant human F-spondin (SPON1), according to the manufacturer’s instructions, and the same amount of PBS was added to the DM group as a control. Cultured cells were maintained under these conditions for 7 days, and mineralization was then assessed by Alizarin Red S staining, and total RNA was extracted for gene expression analysis.

### 2.6. Immunocytochemistry

HDPSCs were seeded at 1 × 10^5^ cells per 3 cm dish and cultured to reach approximately 50% confluence. The cells were then fixed with 4% paraformaldehyde (PFA; Merck Millipore, Darmstadt, Germany) and 0.5% dimethylsulfoxide (Nacalai Tesque) in PBS for 20 min at room temperature. After washing with PBS, the cells were permeabilized with 0.1% Triton X-100 in PBS for 20 min and blocked with 2% bovine serum albumin in PBS for 1 h at room temperature. The cells were incubated overnight at 4 °C with an anti-SPON1 primary antibody (ProSci, Poway, CA, USA; dilution 1:100) or normal rabbit IgG (1:200) as a negative control. The cells were then washed and incubated with Alexa Fluor 568-conjugated goat anti-rabbit IgG secondary antibody (Invitrogen; 1:200) for 1 h at room temperature in the dark. Nuclei were counterstained with 4′,6-diamidino-2-phenylindole (DAPI; Nacalai Tesque) and images were acquired using a Biozero digital microscope (Keyence).

### 2.7. Animal Model and Surgical Procedures

All animal experiments were approved by the Animal Ethics Committee of Kyushu University (protocol A25-431-0) and conducted in accordance with institutional guidelines. Male Wistar rats (8 weeks old, 200–250 g; Kyudo, Saga, Japan) were housed under standard conditions with free access to food and water. General anesthesia was induced by intraperitoneal injection of medetomidine (0.15 mg/kg), butorphanol tartrate (2.5 mg/kg), and midazolam (2 mg/kg). For baseline histological assessment, intact upper molars were harvested following transcardial perfusion with 4% PFA in PBS. Samples were kept overnight at 4 °C and decalcified in 10% ethylenediaminetetraacetic acid (FUJIFILM Wako Pure Chemical Corporation, pH 7.3) at 4 °C for 4–6 weeks before paraffin embedding. To evaluate reparative dentin formation, standardized shallow cavities (depth about 0.6 mm) were prepared on the buccal and mesial surfaces of the upper molars, without pulp exposure, using a round bar (diameter 0.6 mm; MANI, Inc., Tochigi, Japan). Rats were sacrificed at 1, 3, 5, 7, 10, or 14 days post-operation (n = 5 per time point) by transcardial perfusion with 4% PFA. Maxillary tissues were similarly post-fixed, decalcified, and embedded in paraffin for histological and immunohistochemical analyses. The surgical procedure was performed based on previous reports [24,25].

### 2.8. Immunohistochemistry (IHC)

Eight-week-old male Sprague Dawley rats were euthanized, and their upper molars were harvested and fixed in 4% PFA in PBS overnight at 4 °C. The specimens were decalcified in 10% EDTA at 4 °C for 4–6 weeks, followed by paraffin embedding and sectioning at 5 μm thickness. After deparaffinization and rehydration, the sections were blocked with 2% bovine serum albumin (Nacalai Tesque) in PBS for 1 h at room temperature, and then incubated overnight at 4 °C with anti-SPON1 primary antibody (ProSci; 1:100). Negative controls were incubated with normal rabbit IgG (1:200). Following primary antibody incubation, the sections were incubated with biotinylated anti-rabbit IgG secondary antibody (Nichirei Biosciences, Tokyo, Japan) for 30 min at room temperature, followed by treatment with an avidin-peroxidase conjugate (Nichirei Biosciences) for 30 min. Positive staining was visualized using diaminobenzidine (DAB) substrate (Nichirei Biosciences), and nuclei were counterstained with Mayer’s hematoxylin (FUJIFILM Wako Pure Chemical Corporation).

### 2.9. Histological Evaluation

For hematoxylin and eosin staining, tissues were sectioned at a thickness of 5 μm, deparaffinized, rehydrated, and stained with Mayer’s hematoxylin solution (FUJIFILM Wako Pure Chemical Corporation) for 100 s at room temperature, washed with running tap water for 10 min, and then immersed in 0.1% eosin solution (FUJIFILM Wako Pure Chemical Corporation) at room temperature five times for 30 s each. The sections were then washed with running tap water for 1 min and observed under a BX41 microscope.

### 2.10. Neutralization of SPON1 Activity

HDPSCs were seeded at 2 × 10^4^ cells per well in 24-well plates and cultured under the following conditions: normal culture medium (CM), odontoblastic differentiation medium supplemented with normal goat IgG (3 µg/mL; R&D Systems) as a negative control (DM), or odontoblastic differentiation medium supplemented with neutralizing anti-human SPON1 antibody (3 µg/mL; R&D Systems). Cells were maintained at 37 °C in 5% CO_2_ for 7 days, and mineralization was assessed at day 7 by Alizarin Red S staining using a BZ-9000 microscope (Keyence). Mineralized areas were quantified using BZ-X Analyzer Software (Keyence). Total RNA was also extracted after incubation for real-time qRT-PCR analysis, as described above.

### 2.11. Small Interfering RNA Transfection

HDPSCs were transfected with human SPON1 small interfering RNA (siSPON1; MISSION siRNA, SASI_Hs02_00340896; Sigma-Aldrich, St. Louis, MO, USA) or human control siRNA (siControl; MISSION siRNA Universal Negative Control #1, SIC-001-10; Sigma-Aldrich, St. Louis, MO, USA) using Lipofectamine RNAiMAX (Invitrogen, Carlsbad, CA, USA) according to the manufacturer’s instructions. Briefly, HDPSCs were seeded at 2 × 10^4^ cells/well in 24-well plates and cultured until they reached 60–80% confluence. For each well, an siRNA–lipid complex comprising 10 pmol siRNA and 3 µL Lipofectamine RNAiMAX in 50 µL Opti-MEM I (Invitrogen) was prepared and added to the cells in 450 µL Opti-MEM I contain 10% FBS. After 48 h of transfection, SPON1 knockdown efficiency was confirmed by qRT-PCR. For odontoblastic differentiation, siControl- and siSPON1-transfected HDPSCs were cultured in differentiation medium (DM: CM supplemented with 2 mM CaCl_2_) for 7 days, with medium changes every 3 days. After 7 days of differentiation, cells were fixed for Alizarin Red S staining. Total RNA was also extracted for qRT-PCR analysis of odontoblast-related genes.

### 2.12. Western Blotting

HDPSCs (3 × 10^4^ cells/well) were seeded into 24-well plates and cultured to reach approximately 80% confluence. The cells were then incubated in CM, DM, or DM + SPON1 for 5 min, and then lysed in Pierce RIPA buffer (Thermo Fisher Scientific Inc., Waltham, MA, USA) supplemented with 1% protease inhibitor cocktail (Sigma-Aldrich) and 1% phosphatase inhibitor cocktail (Thermo Fisher Scientific Inc.). Approximately 20 μg of protein per lane was separated on 10% sodium dodecyl sulfate–polyacrylamide gels and transferred onto an Immuno-Blot PVDF membrane (Bio-Rad Laboratories, CA, USA). After blocking with 5% skim milk (Yukijirushi, Tokyo, Japan) for 1 h at room temperature, the membrane was incubated with mouse monoclonal anti-β-actin (1:1000; Santa Cruz Biotechnology, Dallas, TX, USA) and rabbit monoclonal anti-non-phospho (active) β-catenin antibody (1:1000; Cell Signaling Technology, Beverly, MA, USA). After detection of active β-catenin, the membrane was stripped and reprobed with rabbit polyclonal anti-total-β-catenin antibody (1:1000; Cell Signaling Technology). These membranes were then incubated with biotinylated anti-rabbit IgG or anti-mouse IgG (Nichirei Biosciences Inc.) for 1 h at room temperature and then reacted with an avidin-peroxidase conjugate (Sigma-Aldrich) for a further hour at room temperature. The membrane was washed thoroughly, and the reactive bands were visualized using ECL Select Western Blotting Detection Reagent (GE Healthcare, Buckinghamshire, UK) and imaged with an ImageQuant LAS 500 system (GE Healthcare).

### 2.13. Statistical Analysis

All experiments were performed using three to five replicates. All values were expressed as mean ± standard deviation. Statistical analysis was performed using one-way ANOVA followed by the Benjamini–Hochberg method. Statistical significance was determined as *p* < 0.05.

## 3. Results

### 3.1. Expression of SPON1 in Rat Dental Pulp Tissue and HDPSCs

**Figure 1:**
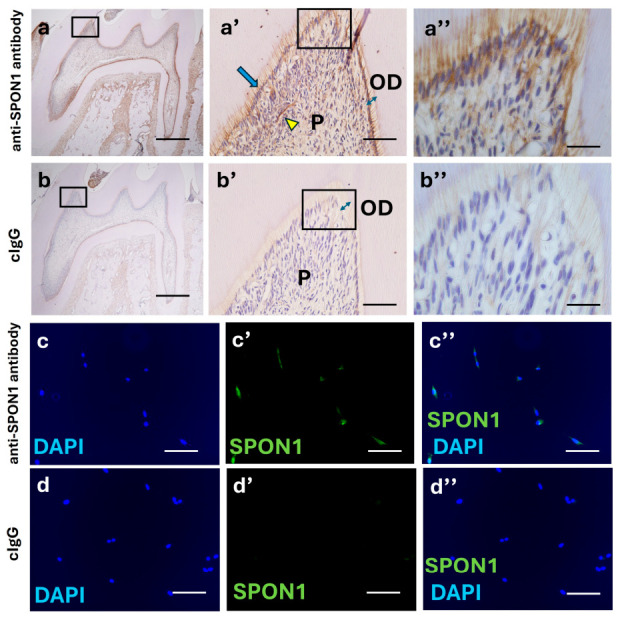
SPON1 expression in dental pulp tissue and cultured HDPSCs (**a**) Immunohistochemical staining of healthy molars from 8-week-old male Wistar rats with anti-SPON1 antibody. SPON1 was expressed in the odontoblast (OD) layer (blue arrow) and dental pulp tissue (yellow arrowhead). Rabbit control IgG (cIgG) served as a negative control (**b**). Higher magnification images of black boxes in panels (**a**,**a′**,**b**,**b′**) are shown in panels (**a′**,**a″**,**b′**,**b″**), respectively. (**c**) Immunocytochemistry of cultured HDPSCs with anti-SPON1 antibody. Rabbit control IgG (cIgG) served as a negative control (**d**). Nuclei stained with DAPI (blue). SPON1 expression was localized in the cytoplasm. Panels (**c″**,**d″**) are merged images of panels c and (**c′**), and panels (**d**,**d′**), respectively. OD, odontoblast layer; P, dental pulp. Scale bars: 500 µm (**a**,**b**); 100 µm (**c**,**d**,**a′**–**d′**,**c″**,**d″**); 20 µm (**a″**,**b″**).

We assessed SPON1 expression levels in rat dental pulp tissue by IHC using an anti-SPON1 antibody. SPON1-positive areas were observed in dental pulp tissue and in the odontoblast layer of the maxillary first molar (Figure 1a). Higher-magnification images further showed SPON1-positive staining in the odontoblast layer and adjacent pulp cells (Figure 1a′,a″). No staining was observed with the IgG control (Figure 1b), supporting the specificity of the staining and endogenous SPON1 expression in vivo.

We then confirmed the endogenous expression of SPON1 in HDPSCs in vitro using immunocytochemistry. Endogenous cytoplasmic localization of SPON1 expression was detected in cultured HDPSCs (Figure 1c). Nuclei were stained blue with DAPI. No staining was observed in the IgG control (Figure 1d).

These findings demonstrated that SPON1 was expressed in the odontoblastic layer and dental pulp tissue of rats in vivo and in the cytoplasm of cultured HDPSCs in vitro.

### 3.2. Expression Pattern of SPON1 During Reparative Dentin Formation

**Figure 2:**
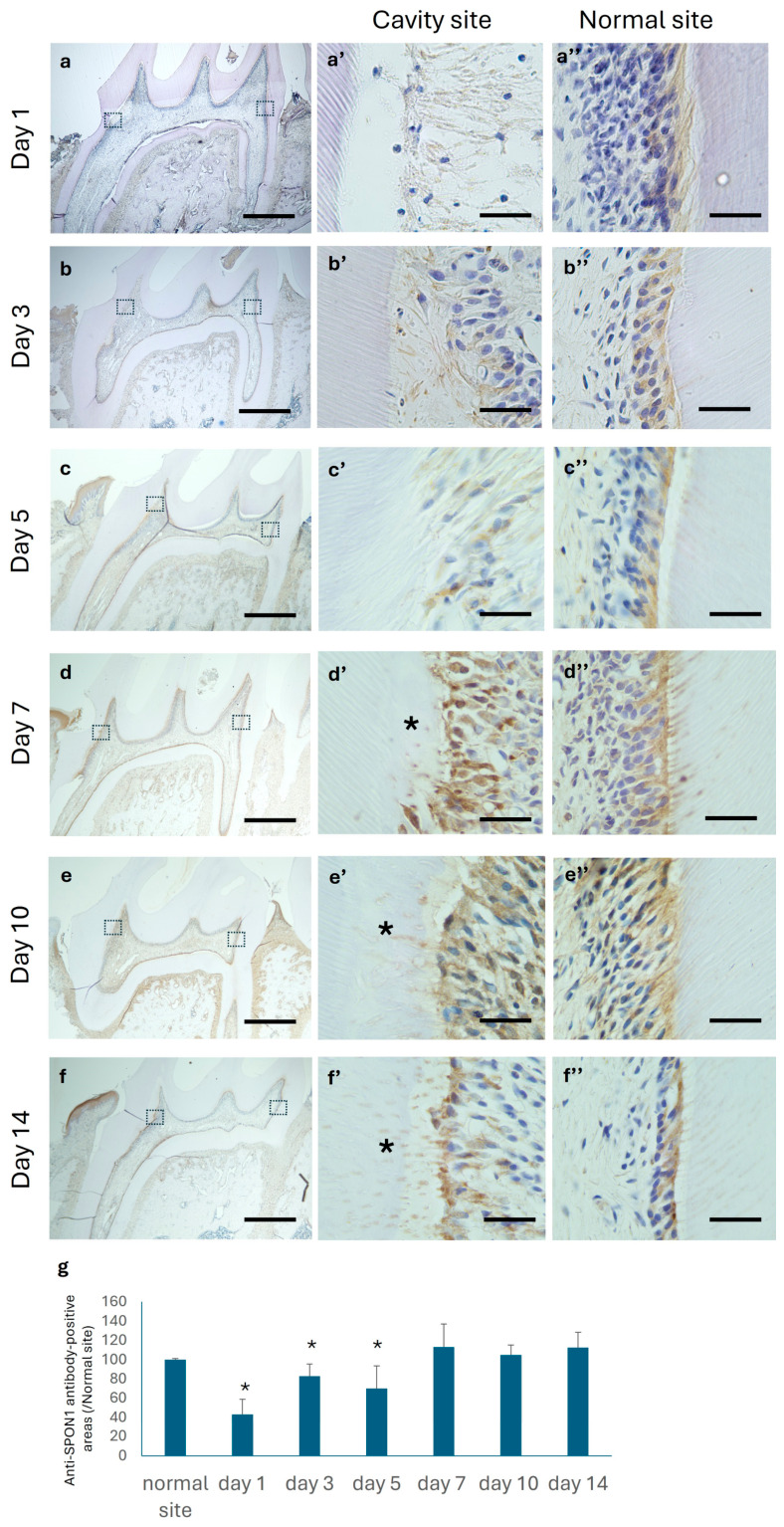
Expression pattern of SPON1 during reparative dentin formation after cavity preparation. (**a**–**f**) Immunohistochemical staining of rat maxillary first molar using anti-SPON1 antibody on days 1, 3, 5, 7, 10, and 14 post-injury. Magnified images of panels (**a**–**f**) shown in panels (**a′**–**f′**) (cavity site; views of injured cavity site) and in panels (**a″**–**f″**) (normal site; views of adjacent healthy pulp tissue), respectively. SPON1 expression was weaker in the cavity site than in the normal site on days 1–5, and became comparable to the control site from day 7 onward, coinciding with reparative dentin formation. Asterisks indicate reparative dentin. (**g**) Quantification of SPON1-positive areas in the odontoblast layer and dental pulp tissue at the cavity site compared with the normal site at different time points after cavity preparation (days 1, 3, 5, 7, 10, and 14). Quantification was performed at ×20 magnification. SPON1-positive areas at the cavity site were normalized to those at the corresponding normal site for each time point. Values are presented as percentages relative to the normal site for each day. * *p* < 0.05, n = 5. Scale bars: 500 µm (**a**–**f**); 20 µm (**a′**–**f′**,**a″**–**f″**).

We analyzed the SPON1 expression pattern during reparative dentin formation in rats by IHC, using an animal model with cavity preparation on the mesial side of the rat upper first molar. A cavity was prepared without pulp exposure, and reparative dentin formation was detected on days 7, 10, and 14 (Figure 2d′–f′). Anti-SPON1 antibody-positive areas were localized in the odontoblastic layer around the cavity sites on days 1, 3, 5, 7, 10, and 14 after surgery (Figure 2a′–f′). Normal sites of maxillary first molars were used as control sites (Figure 2a″–f″). SPON1-positive areas were decreased around the cavity sites on days 1, 3, and 5 after surgery (Figure 2a′–c′), compared with the normal sites (Figure 2a″–c″). There were no differences in anti-SPON1 antibody-positive areas in the odontoblastic layers between cavity sites (Figure 2d′–f′) and normal sites (Figure 2d″–f″) on days 7, 10, and 14 after surgery. Negative controls stained with rabbit control IgG showed no positive staining (Supplementary Figure S1). Quantification of SPON1-positive areas showed fewer SPON1-positive areas in cavity sites on days 1, 3, and 5 compared with normal sites (Figure 2g and Supplementary Figure S2).

These findings suggested that expression of SPON1 was transiently reduced during the early response to cavity preparation and returned to physiological levels during the later stages of reparative dentin formation.

### 3.3. Expression of SPON1 During Odontoblastic Differentiation of HDPSCs

**Figure 3:**
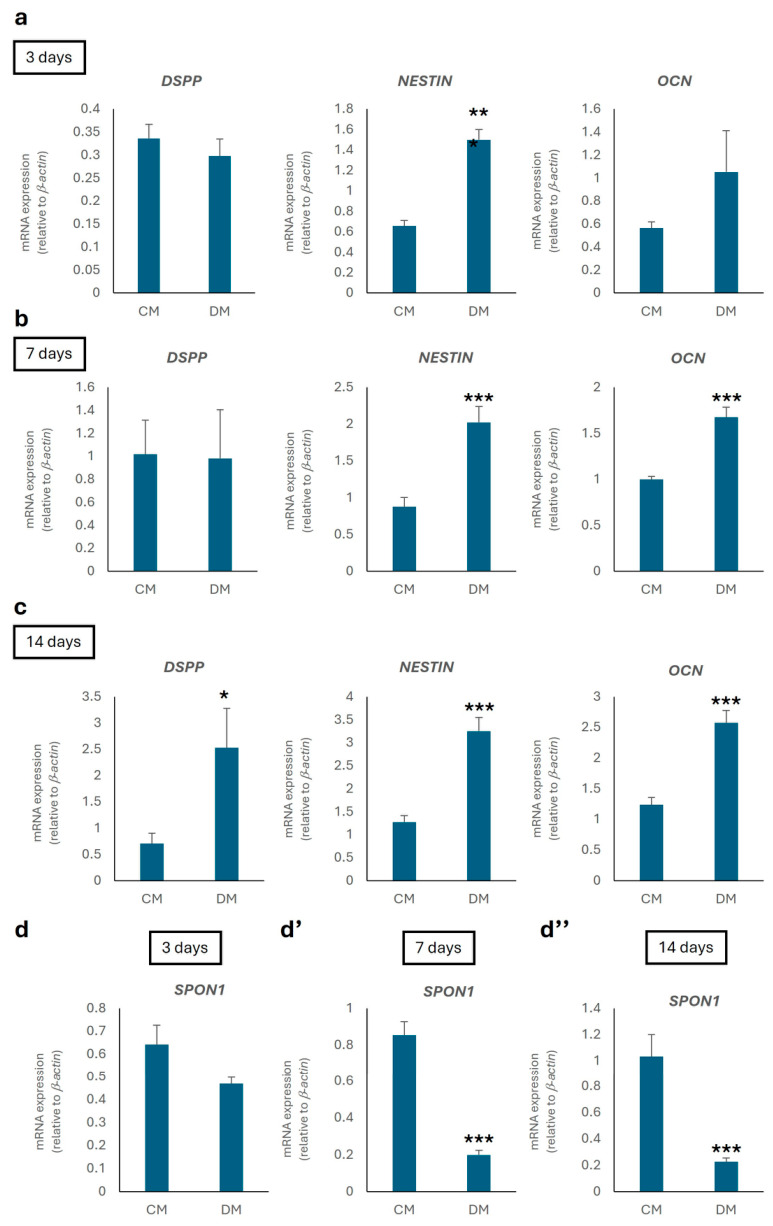
Gene expression profile of *SPON1* during odontoblastic differentiation of HDPSCs (**a**–**c**) qRT-PCR analysis of odontoblast-related markers (*DSPP*, *NESTIN*, and *OCN*) in HDPSCs cultured in 10% FBS/α-MEM control medium (CM) or odontoblastic differentiation-inducing medium (DM = CM + 2 mM CaCl_2_) for 3 (**a**,**d**), 7 (**b**,**d′**) and 14 (**c**,**d″**) days. Time-dependent upregulation of all three markers was observed, indicating progressive odontoblastic differentiation. (**d**) SPON1 expression was progressively downregulated over the same period, suggesting an inverse association with odontogenic commitment. Gene expression levels were normalized to β-actin. Data presented as mean ± standard deviation (n = 3). * *p* < 0.05, ** *p* < 0.01, *** *p* < 0.001 compared with CM at the same time point. DSPP, dentin sialophosphoprotein; OCN, osteocalcin.

We assessed SPON1 expression during odontoblastic differentiation in HDPSCs cultured in CM and DM for different durations (3, 7, and 14 days), and examined the expression of odontoblast-related markers at each stage of the differentiation process. *NESTIN* expression on day 3 was upregulated in the DM group compared with the CM group, but there were no significant changes in *OCN* and *DSPP* expression (Figure 3a), indicating an early stage of differentiation. Accordingly, *NESTIN* and *OCN* expression levels were significantly upregulated by day 7, indicating ongoing differentiation (Figure 3b), and expression levels of all three markers were significantly upregulated on day 14 (Figure 3c), consistent with mature odontoblastic differentiation.

Interestingly, *SPON1* displayed the opposite expression pattern, with slight downregulation in DM on day 3; however, the difference was not significant (Figure 3d). Surprisingly, *SPON1* expression levels were clearly reduced in DM compared with CM on days 7 and 14 (Figure 3d′,d″).

These results indicated that SPON1 expression decreased as odontoblastic differentiation progressed, suggesting an inverse relationship with odontoblast-related markers during odontoblastic differentiation of HDPSCs.

### 3.4. Effects of SPON1 Stimulation on Odontoblastic Differentiation of HDPSCs

We determined the effective concentration of SPON1 for odontoblastic differentiation in HDPSCs cultured in DM with various concentrations of recombinant SPON1 for 7 days. Treatment with recombinant human SPON1 inhibited the formation of mineralized nodules and decreased the Alizarin Red S-positive area in a dose-dependent manner up to 100 ng/mL (Supplemental Figure S3). Based on this result, we used 100 ng/mL recombinant SPON1 protein in subsequent experiments to assess the odontoblastic differentiation of HDPSCs.

**Figure 4:**
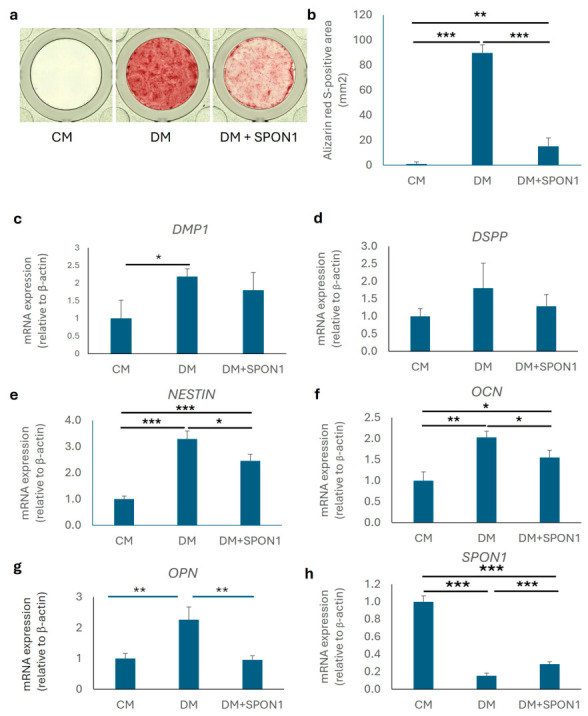
Effect of SPON1 on mineralization and odontoblast-related gene expression in HDPSCs (**a**) Representative images of Alizarin Red S-positive HDPSCs cultured for 7 days in 10% FBS/α-MEM control medium (CM), differentiation medium (DM: CM + 2 mM CaCl_2_), and DM supplemented with SPON1 (DM + 100 ng/mL SPON1). Extensive mineralized nodule formation was observed in DM, while the staining intensity was reduced in the DM + SPON1 group. (**b**) Quantification of Alizarin Red S-positive areas. Mineralization was significantly reduced in the DM + SPON1 group compared with the DM group. (**c**–**g**) Expression levels of odontoblast-related genes (*DMP1*, *DSPP*, *NESTIN*, *OCN* and *OPN*) were analyzed by qRT-PCR. Odontogenic markers were downregulated in the DM + SPON1 group compared with the DM group. (**h**) *SPON1* expression analyzed by qRT-PCR. Data presented as mean ± standard deviation (n = 3). * *p* < 0.05, ** *p* < 0.01, *** *p* < 0.001 vs. DM. DSPP, dentin sialophosphoprotein; OCN, osteocalcin; OPN, osteopontin; DMP1, dentin matrix protein 1.

Cells were cultured in CM, DM, or DM supplemented with 100 ng/mL SPON1 (DM + SPON1) for 7 days. Cells in the DM + SPON1 group generated fewer nodules than the DM group (Figure 4a) and the Alizarin Red S-positive area was significantly reduced in the DM + SPON1 group compared with the DM group (Figure 4b).

We further assessed the role of recombinant SPON1 by detecting the effect of SPON1 stimulation on gene expression of odontogenic markers. DM HDPSCs showed increased expression of *DMP1*, *DSPP*, *NESTIN*, *OCN* and *OPN* compared with the CM group. In contrast, all these markers were downregulated in the DM + SPON1 group, suggesting that SPON1 negatively regulated both mineralization and odontoblast-related gene expression (Figure 4c–f). Endogenous SPON1 levels were also highest in the CM group and strongly decreased in the DM group (Figure 4g), while exogenous SPON1 slightly raised *SPON1* mRNA levels (Figure 4g). The results of immunocytochemistry revealed that expression of DSP and NESTIN was downregulated in the DM + SPON1 group compared with DM group (Supplementary Figure S4). These results confirmed that exogenous SPON1 suppressed mineralized nodule formation and downregulated key odontoblast-related genes.

### 3.5. Effect of SPON1 Neutralization on Odontoblastic Differentiation of HDPSCs

**Figure 5:**
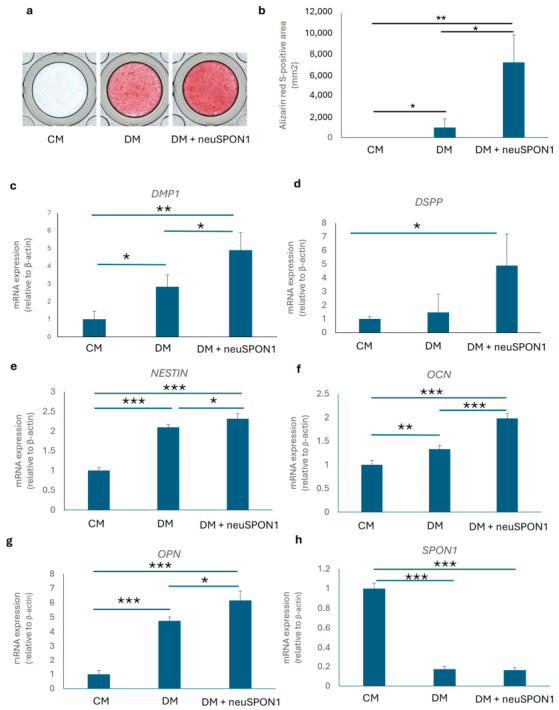
Neutralization of SPON1 promoted mineralization of HDPSCs (**a**) Representative images of Alizarin Red S-positive HDPSCs cultured for 7 days in control medium (CM), differentiation medium (DM: CM + 2 mM CaCl_2_), and DM supplemented with 3 µg/mL anti-SPON1 antibody (DM + nSPON1). Mineralized nodule formation was more intense in the DM + nSPON1 group compared with the DM group. (**b**) Quantification of Alizarin Red S-positive areas. Mineralization was significantly higher in the DM + nSPON1 group compared with the DM group. (**c**–**g**) Expression of odontoblast-related genes (*DMP1*, *DSPP*, *NESTIN*, *OCN* and *OPN*) analyzed by qRT-PCR. (**h**) *SPON1* expression analyzed by qRT-PCR. Data presented as mean ± standard deviation. n = 3. * *p* < 0.05, ** *p* < 0.01, *** *p* < 0.001. DSPP, dentin sialophosphoprotein; OCN, osteocalcin; OPN, osteopontin.

We blocked SPON1 activity during odontoblastic differentiation by culturing HDPSCs in CM, DM, and DM supplemented with a SPON1-neutralizing antibody (DM + neuSPON1) for 7 days. Cells in the DM + neuSPON1 group exhibited significantly higher Alizarin Red S-positive areas compared with the DM group (Figure 5a,b). 

We then analyzed the gene expression levels of odontoblast-related markers under the same conditions. Cells in the DM group showed increased expression levels of *DMP1*, *DSPP*, *NESTIN*, *OCN* and *OPN* compared with the CM group. Cells in the DM + neuSPON1 group also showed significantly enhanced expression of these genes compared with the DM group, consistent with the Alizarin Red S staining results (Figure 5c–g). These findings indicated that neutralizing SPON1 promoted mineralization and the expression of odontoblast-related markers in HDPSCs, suggesting that SPON1 may inhibit odontoblastic differentiation of HDPSCs.

### 3.6. Effects of SPON1 Knockdown on Odontoblastic Differentiation of HDPSCs

**Figure 6:**
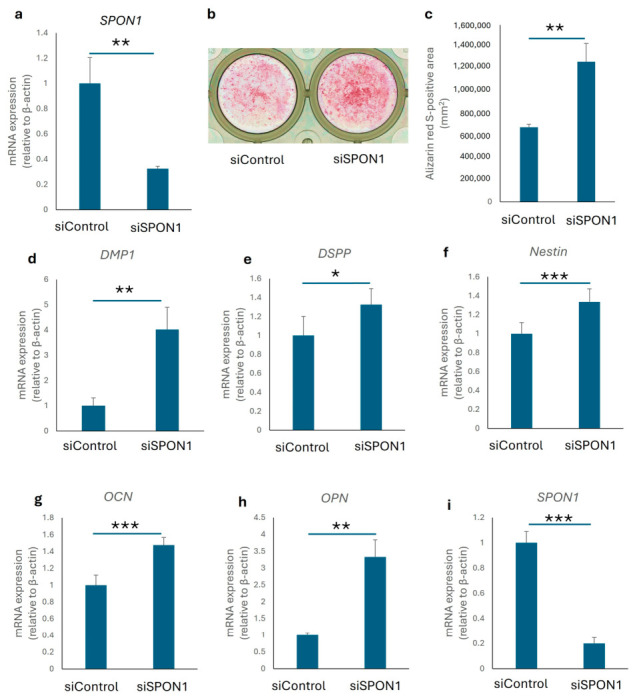
SPON1 knockdown enhanced odontoblastic differentiation of HDPSCs. (**a**) SPON1 knockdown efficiency was confirmed by qRT-PCR 48 h after transfection with siControl and siSPON1. SPON1 mRNA expression was significantly reduced in the siSPON1 group compared with the siControl group. (**b**) Representative images of Alizarin Red S-positive HDPSCs after 48 h of siRNA transfection followed by 7 days of culture in differentiation medium (DM: CM + 2 mM CaCl_2_). Alizarin red S-positive area was more intense in the siSPON1 group compared with the siControl group. (**c**) Quantification of Alizarin Red S-positive areas. The siSPON1 group showed significantly higher mineralization than the siControl group. (**d**–**h**) Expression levels of odontoblast-related genes *DSPP*, *NESTIN*, *OCN*, *OPN*, and *DMP1* analyzed by qRT-PCR after 48 h of siRNA transfection, followed by 7 days of culture in DM. (**i**) SPON1 expression remained significantly reduced in the siSPON1 group after differentiation culture. Data are presented as mean ± standard deviation (n = 4). * *p* < 0.05, ** *p* < 0.01, *** *p* < 0.001. DSPP, dentin sialophosphoprotein; OCN, osteocalcin; OPN, osteopontin; DMP1, dentin matrix protein 1.

We suppressed SPON1 expression in HDPSCs using siRNA to further examine the role of endogenous SPON1 during odontoblastic differentiation. SPON1 knockdown was confirmed 48 h after transfection, and cells in the siSPON1 group showed significantly lower SPON1 mRNA expression compared with the siControl group (Figure 6a). After 48 h of transfection, cells were cultured in DM for 7 days. Cells in the siSPON1 group exhibited stronger Alizarin Red S staining and significantly higher Alizarin Red S-positive areas compared with the siControl group (Figure 6b,c). We then analyzed the gene expression levels of odontoblast-related markers under the same conditions. Cells in the siSPON1 group showed significantly enhanced expression of *DSPP*, *NESTIN*, *OCN*, *OPN*, and *DMP1* compared with the siControl group (Figure 6d–h), while SPON1 expression remained significantly reduced after differentiation culture (Figure 6i). These findings indicated that SPON1 knockdown promoted mineralization and the expression of odontoblast-related markers in HDPSCs, further suggesting that endogenous SPON1 may inhibit odontoblastic differentiation of HDPSCs.

### 3.7. Effects of SPON1 on Wnt/β-Catenin Signaling Pathway During Odontoblastic Differentiation of HDPSCs

**Figure 7:**
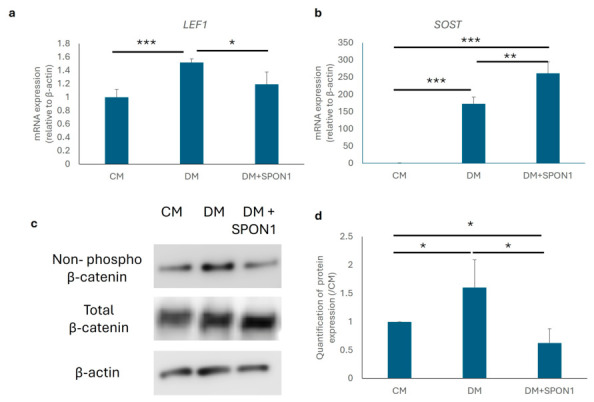
Effects of SPON1 on Wnt-β-catenin signaling during odontoblastic differentiation of HDPSCs (**a**,**b**) Expression levels of Wnt-β-catenin signal-related genes (*LEF1* and *SOST*) analyzed by qRT-PCR. SPON1 treatment (DM + SPON1) led to downregulation of *LEF1* and upregulation of *SOST* compared with the DM group. Data presented as mean ± standard deviation (n = 3). (**c**) Western blot analysis to detect active non-phosphorylated-β-catenin and total-β-catenin in HDPSCs cultured in 10% FBS/α-MEM control medium (CM), differentiation medium (DM: CM + 2 mM CaCl_2_), and DM supplemented with SPON1 (DM + 100 ng/mL SPON1) for 5 min. β-actin used as a loading control. Original images of Western blotting in Figure 7 were shown in Supplementary Figure S5. (**d**) Quantification of active/total β-catenin ratio after normalization to β-actin. * *p* < 0.05, ** *p* < 0.01, *** *p* < 0.001 vs. DM. LEF1, lymphoid enhancer-binding factor 1; SOST, sclerostin.

Recent studies reported that canonical Wnt/β-catenin signaling plays an essential role in reparative dentin formation [16,17] and tooth development [18]. We investigated whether SPON1 modulated Wnt/β-catenin signaling during odontoblastic differentiation by analyzing the expression of Wnt target genes by qRT-PCR. Cells were cultured in CM, DM, or DM supplemented with 100 ng/mL SPON1 (DM + SPON1). *LEF1* expression was significantly upregulated in the DM group compared with the CM group, but was downregulated in the DM + SPON1 group compared with the DM group (Figure 7a). Expression of *SOST*, an inhibitor of Wnt/β-catenin signaling, was significantly increased in the DM + SPON1 group compared with the DM group (Figure 7b).

We also examined the effects of SPON1 on the levels of active non-phosphorylated β-catenin in HDPSCs. Western blotting analysis showed that the levels of active non-phosphorylated β-catenin were significantly increased in the DM group compared with the CM group, but these levels were significantly decreased in the DM + SPON1 group compared with the DM group (Figure 7c,d). The band intensity was quantified and normalized to total β-catenin. Original images of Western blotting analyses are shown in Supplementary Figure S5. These findings suggested that Wnt/β-catenin signaling was involved in odontoblastic differentiation and that SPON1 treatment was associated with suppression of this activation.

## 4. Discussion

Reparative dentin forms a protective barrier in response to a variety of pathological injuries and is formed by odontoblast-like cells, which are derived from DPSCs [26]. However, reparative dentin is self-limited, and its deposition must be restricted to the injured area to avoid pathological calcification or pulp canal obliteration. Dental pulp healing depends on the coordinated activation of reparative dentin formation while preserving tissue homeostasis and preventing excessive mineral deposition. Although numerous studies have focused on the signals that promote odontoblast-like cell differentiation [27,28], less attention has been paid to the factors that limit, inhibit, or regulate mineralization during reparative dentin formation. Some previous studies showed the existence of inhibitory regulators of odontoblastic differentiation of DPSCs [29,30], but the detailed mechanism has not been revealed. SPON1 has been reported to be involved in bone metabolism: one study found that *Spon1*-knockout mice showed a high bone-mass phenotype. Based on these observations and the shared molecular pathways and features between bone and dentin [31], we investigated SPON1 as a potential regulator of HDPSC differentiation.

We found that SPON1 was expressed in the odontoblastic layer and dental pulp tissue in healthy rat molars, and showed endogenous cytoplasmic localization in HDPSCs. Although SPON1 is an ECM protein, its predominantly cytoplasmic localization in cells is consistent with intracellular synthesis, trafficking, or processing before secretion. The current findings were partly supported by previous studies demonstrating SPON1 expression in dental tissues, particularly in the periodontal ligament [32,33] and cementoblasts [13,19]; however, to the best of our knowledge, the current results provide the first indication of SPON1 expression in the odontoblast layer and its possible association with reparative dentin formation.

Tooth drilling is one of the most frequent clinical dental treatments, and acute and chronic pulp responses to cavity preparation have therefore attracted considerable research attention. Cavity preparation causes both the dentin and pulp to undergo substantial morphological changes, which can be explained by the fact that the full thickness of the dentin is traversed by the cytoplasmic processes of odontoblasts [34]. Particularly in deep cavity preparation models, early odontoblastic injury is followed by the appearance of new odontoblast-like cells and tertiary dentin deposition, consistent with the formation of reparative dentin rather than reactionary dentin formed by surviving odontoblasts [25]. One day after cavity preparation, many odontoblasts separated from the predentin, followed by degeneration, and repair of the destroyed odontoblastic layer started 3 days after cavity preparation, as spindle-shaped cells presumably corresponding to pre-odontoblasts replaced the damaged odontoblasts and became mature enough to produce reparative dentin by days 5–7. The new odontoblasts underlying the reparative dentin were no longer distinguishable from the adjacent intact odontoblasts by 10–15 days. This is consistent with a more stabilized repair phase at this stage than on days 3–7 [34,35].

We investigated the involvement of SPON1 in reparative dentin formation in vivo using a deep cavity-preparation model [25]. We observed a localized transient reduction in SPON1 immunoreactivity in the cavity area, which was significantly weaker at days 1–5 compared with the normal area of the same tooth. This decrease in SPON1-positive area was unlikely to be explained solely by the loss of injured odontoblasts, because the reduction was still evident at days 3 and 5, when odontoblast-like cells were replacing the damaged odontoblasts and reparative dentin formation had already begun. SPON1 immunoreactivity was increased by day 7 and approached normal levels by days 10 and 14. Overall, the current results suggest that SPON1 was transiently reduced early to permit reparative dentin formation and subsequently restored to normal levels after the repair process was almost finished.

We analyzed the expression levels of *DSPP*, *DMP1*, *NESTIN*, *OCN*, and *OPN*, as common markers of odontoblastic differentiation in HDPSCs, in an in vitro model. We previously demonstrated that stimulation with CaCl_2_ induced the differentiation of HDPSCs into odontoblast-like cells [22,36]. The earlier increase in *NESTIN*, followed by later upregulation of *OCN*, *OPN*, *DMP1* and *DSPP*, was consistent with temporal progression towards an odontoblastic phenotype. In contrast, SPON1 levels were not increased during the early stage and became significantly downregulated when all the markers were upregulated. This relationship suggests an inverse association between SPON1 expression and odontoblastic differentiation in HDPSCs.

Various signals, such as growth factors, chemokines, and ECMs, are involved in the regeneration and homeostasis of the dentin–pulp complex [37]. We therefore investigated the effects of recombinant SPON1 protein on the odontoblastic differentiation of HDPSCs. We confirmed that recombinant SPON1-treated cells formed fewer mineralized nodules and showed lower expression of odontoblast-related markers, such as *DSPP*, *NESTIN*, *OCN*, *OPN*, and *DMP1*, compared with untreated cells. This was consistent with a previous report that found that exogenous SPON1 inhibited the longitudinal growth of mouse tibiae [14].

Consistent with the inhibitory effect of recombinant SPON1 on the odontoblastic differentiation of HDPSCs, neutralization of endogenous SPON1 enhanced mineralized nodule formation and the expression of odontoblast-related markers, including *DSPP*, *NESTIN*, *OCN*, *OPN*, and *DMP1*, in HDPSCs. Importantly, the neutralizing antibody is expected to block SPON1 protein activity rather than directly suppress SPON1 transcription. Therefore, enhanced mineralization in the DM + neuSPON1 group may occur even when SPON1 mRNA expression is not markedly different from that in the DM group. This suggests that residual extracellular or cell-associated SPON1 protein may still exert an inhibitory effect during odontoblastic differentiation, and that blocking this activity can further promote mineralization. These findings are in line with a previous report demonstrating that *Spon1*-knockout mice showed high bone mass compared with wild-type mice [15]. In addition to antibody-mediated neutralization, siRNA-mediated SPON1 knockdown further supported the inhibitory role of endogenous SPON1. SPON1 knockdown significantly enhanced Alizarin Red S-positive mineralization and increased the expression of DSPP, NESTIN, OCN, OPN, and DMP1 under odontoblastic differentiation conditions. These findings strengthen the conclusion that SPON1 inhibits odontoblastic differentiation, because enhanced differentiation was observed not only after blocking SPON1 activity with a neutralizing antibody, but also after reducing endogenous SPON1 expression by siRNA. Overall, the gain-of-function and loss-of-function approaches indicated that SPON1 acts as a negative regulator of odontoblastic differentiation of HDPSCs and may function as a brake during reparative dentin formation.

Previous reports demonstrated that β-catenin was expressed in odontoblast-like cells lining the inner surface of reparative dentin [16,38], and canonical Wnt/β-catenin signaling promoted the proliferation and odontoblastic differentiation of stem cells derived from the apical papilla [39]. In addition, elevated extracellular Ca^2+^ can function as a signaling ion through the calcium-sensing receptor and engage in crosstalk with Wnt pathways, including modulation of β-catenin-dependent transcription [40,41]. Several previous studies reported that SPON1 signaling functioned through low-density lipoprotein receptor-related protein 8 [12,19], which is a known receptor of Wnt/β-catenin signaling during osteoblastic differentiation [42]. We therefore hypothesized that canonical Wnt/β-catenin signaling may play a role in mediating the effects of SPON1 on odontoblastic differentiation of HDPSCs. Levels of stabilized non-phosphorylated β-catenin were increased in CaCl_2_-treated cells compared with untreated cells, but levels were decreased in CaCl_2_ + SPON1-treated cells. Consistent with these results, CaCl_2_ also altered canonical Wnt transcriptional readouts, including *LEF1*, and the reverse effect was observed in CaCl_2_ + SPON1-treated cells. SOST, a secreted antagonist of Wnt/β-catenin signaling [43], was significantly upregulated in CaCl_2_ + SPON1-treated cells, consistent with an increased inhibitory signaling environment. However, CaCl_2_-treated cells also showed upregulated SOST expression, suggesting feedback inhibition triggered by Wnt/β-catenin activation during odontoblastic differentiation. These findings suggest that SPON1-mediated inhibition of odontoblastic differentiation is associated, at least in part, with suppression of canonical Wnt/β-catenin signaling in HDPSCs. However, further studies including pathway-specific inhibition or rescue are needed to confirm these results. Furthermore, this study only focused on the Wnt/β-catenin signaling pathway. Thus, it will be necessary to consider and analyze other signals involved in regulating restorative dentin formation, such as TGF-β signaling [44].

Overall, our findings support the role of SPON1 as a negative regulator of odontoblastic differentiation of DPSCs. This conclusion is supported by the transient reduction in SPON1 during the early stage of reparative dentin formation in vivo, the inverse association between SPON1 and odontoblast-related markers during HDPSC differentiation, the inhibitory effect of recombinant SPON1, and the enhanced differentiation observed after SPON1 neutralization and siRNA-mediated SPON1 knockdown. These findings suggest that SPON1 may regulate reparative dentin formation to avoid excessive calcification and pulp canal obliteration. Furthermore, neutralizing SPON1 activity may have therapeutic potential for use in direct pulp-capping materials. However, this study has some limitations. Although SPON1 expression was analyzed in vivo, the functional effects of SPON1 were mainly examined in vitro, which may not fully reflect the in vivo microenvironment of dental pulp tissue. In addition, although we identified SPON1 as a potential negative regulator of reparative dentin formation, the precise molecular mechanisms remain unclear. Therefore, further studies will be necessary to determine the mechanism of regulating reparative dentin formation.

## 5. Conclusions

SPON1 was expressed in the odontoblastic layer and dental pulp tissue in rats and in the cytoplasm of cultured HDPSCs. In addition, SPON1 expression was decreased at cavity sites during the early stage of reparative dentin formation compared with normal sites in a rat cavity preparation model. Exogenous SPON1 suppressed odontoblastic differentiation of HDPSCs in vitro, whereas SPON1 neutralization and siRNA-mediated SPON1 knockdown enhanced mineralization and odontoblast-related marker expression, indicating an inverse association between SPON1 and odontoblastic differentiation of HDPSCs. Mechanistically, this inhibitory effect appeared to be mediated, at least in part, through suppression of canonical Wnt/β-catenin signaling. Overall, the current findings suggest that SPON1 acts as a negative regulator of odontoblastic differentiation in HDPSCs. These findings provide new insights into the molecular mechanisms underlying reparative dentin formation and suggest that direct pulp-capping materials designed to neutralize SPON1 activity may represent a promising strategy for dental pulp preservation.

## Data Availability

The original contributions presented in this study are included in the article/Supplementary Materials; further inquiries can be directed to the corresponding author.

## Supplementary Materials

The following supporting information can be downloaded at: https://www.mdpi.com/article/10.3390/biom16060769/s1, Figure S1: Negative control IgG staining during reparative dentin formation after cavity preparation in rats; Figure S2: Comparison of SPON1 expression between the cavity and normal sites during reparative dentin formation after cavity preparation day by day; Figure S3: Dose-dependent effect of recombinant SPON1 on mineralization of HDPSCs; Figure S4: Expression of odontoblast-related markers in SPON1-treated HDPSCs; Figure S5: Original images of western blotting analyses; Supplementary Table: Primer sequence, product size, and annealing temperature for quantitative RT-PCR.
