# Supplementary material for: Spondin-1 Inhibits Odontoblastic Differentiation of Human Dental Pulp Stem Cells
Source: Biomolecules. 2026 May 23;16(6):769. doi: 10.3390/biom16060769 (PMC13297351; doi:10.3390/biom16060769)
Supplement: Supplementary file 1 [file biomolecules-16-00769-s001.zip › biomolecules-4289459-supplementary.pdf]

Supplementary Figures and Table

Target gene	Forward primer (5'→3')	Reverse primer (5'→3')	Annealing temp (°C)	Cycles	Product size (bp)
DSPP	ATATTGAGGGCTGGAATGGGGA	TTTGTGGCTCCAGCATTGTCA	60	40	136
Nestin	TGGCCACGTACAGGACCCTCC	AGATCCAAGACGCCGGCCCT	60	40	143
OCN	GTGCAGAGTCCAGCAAAGGT	TCAGCCAACTCGTCACAGTC	60	40	175
OPN	ACACATATGATGGCCGAGGTGA	TGTGAGGTGATGTCCTCGTCTGT	60	40	115
DMP1	CCCTTGGAGAGCAGTGAGTC	CTCCTTTTCCTGTGCTCCTG	60	40	166
SPON1	CCCAAGTCAGAGGGCTACTG	GGTTCCCGGCTTGTAGAAGT	60	40	118
LEF1	CTACCCATCCTCACTGTCAGTC	GGATGTTCCTGTTTGACCTGAGG	60	40	135
SOST	TTCTCCTTCGGGACCTCAAT	TCTCTCACCTCTGCCCATTC	60	40	126
β-actin	ATTGCCGACAGGATGCAGA	GAGTACTTGCGCTCAGGAGGA	60	40	89

Supplementary Table. Primer sequence, product size, and annealing temperature for quantitative RT-PCR

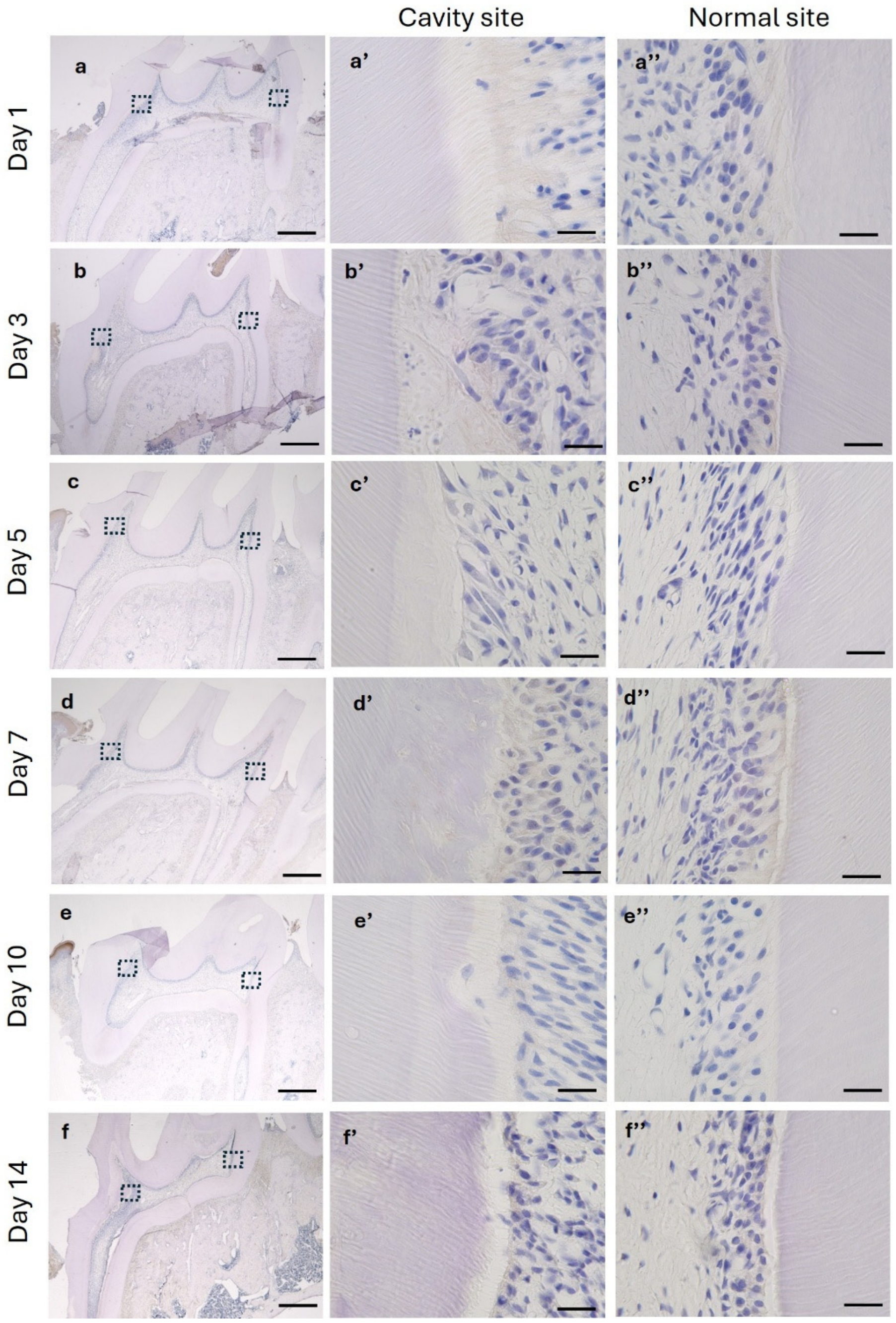

Supplementary Fig. 1 Negative control IgG staining during reparative dentin formation after cavity preparation in rats

(a–f) Immunohistochemical staining of rat maxillary first molar using a control IgG antibody on days 1, 3, 5, 7, 10, and 14 post-injury. Magnified images of panels a–f are shown in panels a'–f' (cavity site) and in panels a''–f'' (normal site). No positive staining was observed in either the cavity site or the normal site at any time point. Scale bars: 500 μm (a–f); 20 μm (a'–f', a''–f'').

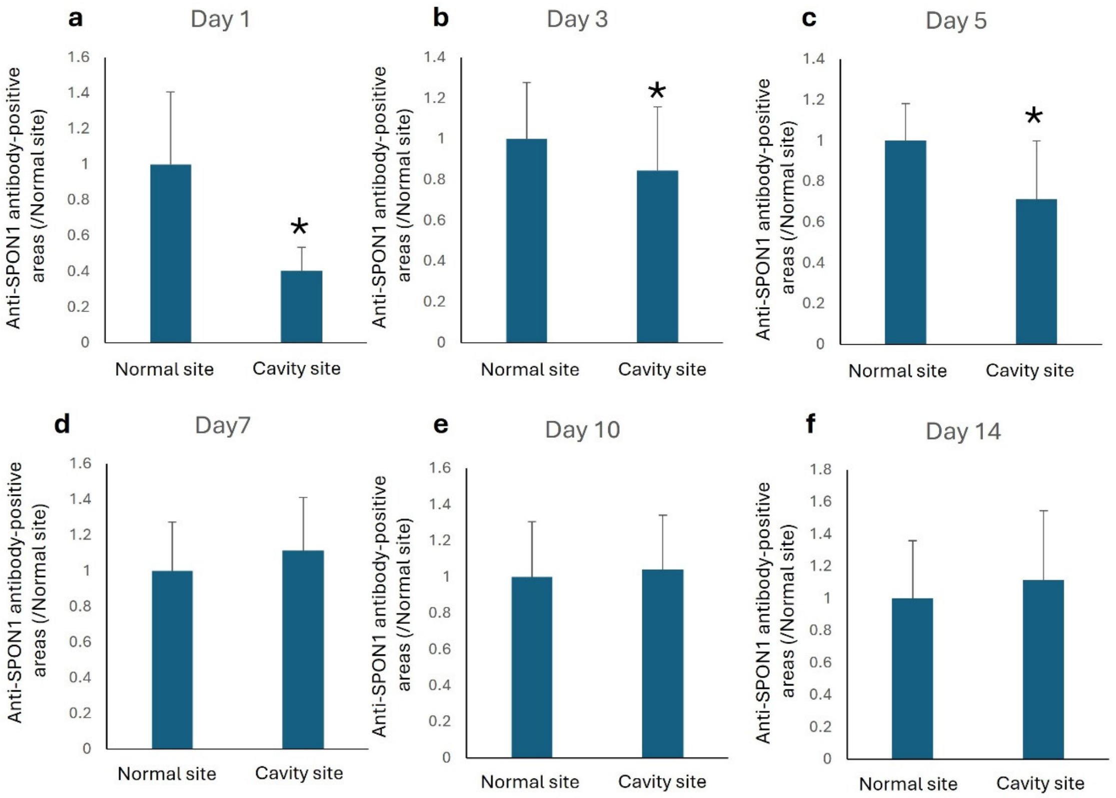

Supplementary Fig. 2 Comparison of SPON1 expression between the cavity and normal sites during reparative dentin formation after cavity preparation day by day
(a–f) Quantification of anti-SPON1 antibody-positive areas in the cavity and normal sites of rat maxillary first molars on days 1, 3, 5, 7, 10, and 14 after cavity preparation. Quantification was performed under ×40 magnification. Values are presented as fold change against Normal sites at each day. Anti-SPON1 antibody-positive area was significantly decreased in cavity site on days 1, 3, and 5, whereas there were no significant differences between cavity and normal sites on days 7, 10, and 14. * $p < 0.05$, $n = 5$.

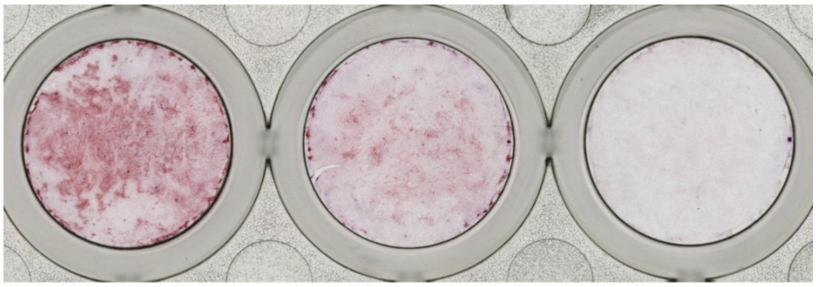

SPON1 10 ng/mL 50 ng /mL 100 ng/mL

Supplementary Fig. 3 Dose-dependent effect of recombinant SPON1 on mineralization of HDPSCs

Images of Alizarin Red S staining of HDPSCs cultures for 5 days with 10% FBS/α-MEM with 2 mM $CaCl_2$ supplemented with SPON1 under three different concentrations (10, 50, and 100 ng/mL). SPON1 inhibited the mineralization of HPDSCs in a dose-dependent manner.

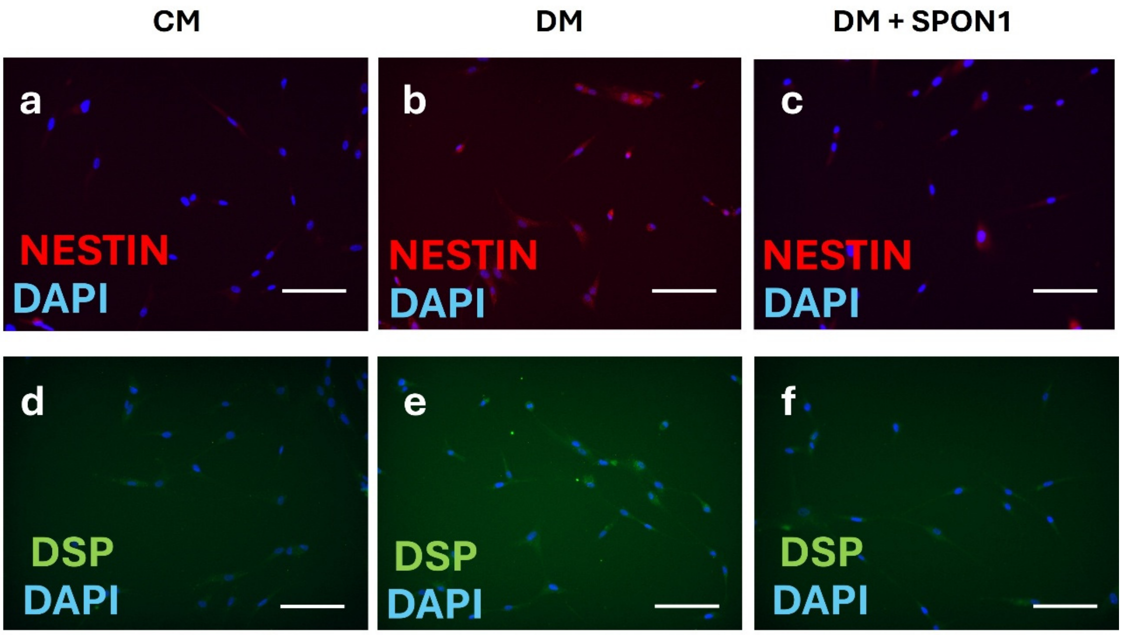

Supplementary Fig. 4 Expression of odontoblast-related markers in SPON1-treated HDPSCs

Immunocytochemistry of cultured HDPSCs with anti-NESTIN antibody (a-c; red) and anti-DSP antibody (d-f; green). HDPSCs were cultured in 10% FBS/α-MEM control medium (CM), differentiation medium (DM: CM + 2 mM $CaCl_2$), and DM supplemented with SPON1 (DM + 100 ng/mL SPON1). Nuclei stained with DAPI (blue). expression of DSP and NESTIN was downregulated in the DM + SPON1 group compared with DM group. Scale bars: 100 µm.

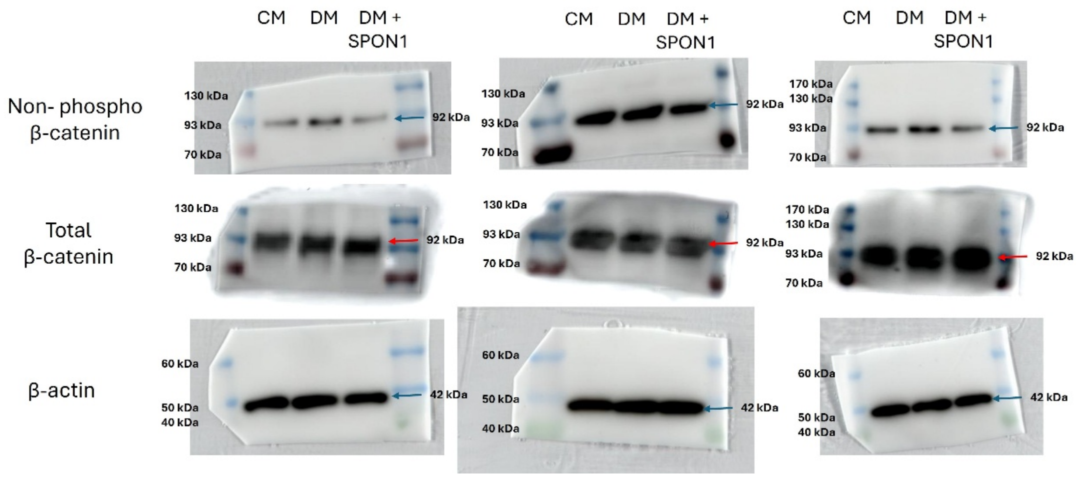

Supplementary Fig. 5 Original images of western blotting analyses

Original images of western blotting in Fig. 6 were shown. The blots of an anti-non-phospho β-catenin antibody (92 kDa), an anti-total β-catenin antibody (92 kDa) and an anti-β-actin antibody (C; 42 kDa) were exhibited (n = 3).
